# Revealing Differences in Metabolic Flux Distributions between a Mutant Strain and Its Parent Strain *Gluconacetobacter xylinus* CGMCC 2955

**DOI:** 10.1371/journal.pone.0098772

**Published:** 2014-06-05

**Authors:** Cheng Zhong, Fei Li, Miao Liu, Xiao-Ning Yang, Hui-Xia Zhu, Yuan-Yuan Jia, Shi-Ru Jia, Luciano Piergiovanni

**Affiliations:** 1 Key Laboratory of Industrial Fermentation Microbiology, (Ministry of Education), Tianjin University of Science & Technology, Tianjin, People’s Republic of China; 2 DeFENS, Department of Food Environmental and Nutritional Sciences, University of Milan, Milan, Italy; 3 Key Laboratory of Systems Bioengineering, Ministry of Education, Tianjin University, Tianjin, People’s Republic of China; University of Groningen, Netherlands

## Abstract

A better understanding of metabolic fluxes is important for manipulating microbial metabolism toward desired end products, or away from undesirable by-products. A mutant strain, *Gluconacetobacter xylinus* AX2-16, was obtained by combined chemical mutation of the parent strain (*G. xylinus* CGMCC 2955) using DEC (diethyl sulfate) and LiCl. The highest bacterial cellulose production for this mutant was obtained at about 11.75 g/L, which was an increase of 62% compared with that by the parent strain. In contrast, gluconic acid (the main byproduct) concentration was only 5.71 g/L for mutant strain, which was 55.7% lower than that of parent strain. Metabolic flux analysis indicated that 40.1% of the carbon source was transformed to bacterial cellulose in mutant strain, compared with 24.2% for parent strain. Only 32.7% and 4.0% of the carbon source were converted into gluconic acid and acetic acid in mutant strain, compared with 58.5% and 9.5% of that in parent strain. In addition, a higher flux of tricarboxylic acid (TCA) cycle was obtained in mutant strain (57.0%) compared with parent strain (17.0%). It was also indicated from the flux analysis that more ATP was produced in mutant strain from pentose phosphate pathway (PPP) and TCA cycle. The enzymatic activity of succinate dehydrogenase (SDH), which is one of the key enzymes in TCA cycle, was 1.65-fold higher in mutant strain than that in parent strain at the end of culture. It was further validated by the measurement of ATPase that 3.53–6.41 fold higher enzymatic activity was obtained from mutant strain compared with parent strain.

## Introduction

Plant-based cellulose, due to its abundance and low-cost, has attracted increasing attention in recent years [1], [2]. However, the presence of lignin, hemicelluloses and other molecules in lignocellulosic biomass makes it complicated processes for medical use [1], [3]. Bacterial cellulose (BC) is an insoluble, extracellular polysaccharide that is produced by certain types of microorganisms, such as *Acetobacter* species [4]. It is a highly pure form of cellulose with a fine nano-scale structure and has been widely used in food and biomedical fields for various applications [5], [6], [7]. BC has the same chemical structure as plant cellulose, but exhibits superior physical and chemical properties, including high mechanical tensile strength, purity, biodegradability and water-holding capacity [3], [5], [7].

Recently, investigations on developing cost-effective culture processes have been broadly carried out using *Gluconacetobacter*, *Acetobacter* etc., to obtain maximum productivity of BC [8], [9]. Till now, increasing BC yield is still a big challenge which limits its further application in various fields [10]. Bae and Shoda [11] reported that adding molasses at a lower concentration of 20 g/L into the cultivation medium improved the efficiency and the econometrics. Jung *et al*. [12] used glycerol as the sole carbon source as opposed to glucose, after seven days of cultivation, BC yield and crystallinity index were 380% and 9% higher, respectively. It has also been reported that cellulose production by *G. xylinus* on glucose medium was enhanced in batch culture when ethanol was present in the media [13]. However, there is limited knowledge regarding the metabolism of existing strains of *Gluconacetobacter*, which makes it difficult to understand the metabolic network and relate it to the production of interesting products (such as acetic acid, cellulose, etc.) [14], [15].

Metabolic flux analysis provides theoretical foundations for analyzing and understanding BC biosynthesis pathway and extra- and intracellular metabolism at specific conditions [16]. In an effort to obtain highly productive strains, researchers have applied genetic engineering methods combined with metabolic flux analysis to adjust or shift the desired metabolic pathway toward desirable products [17]. Velasco-Bedrán and Lopezsunza-Isunza [14] analyzed the catabolic and anabolic pathway for *Gluconacetobacter* central metabolism, but the metabolic fluxes towards biomass accumulation were not taken into consideration. In one of our recent researches, the metabolic network in *Gluconacetobacter xylinus* CGMCC 2955 was constructed, and the metabolic flux distributions in this strain cultured on three different carbon sources (glucose, fructose and glycerol) were compared [15]. It has been revealed that byproducts accumulation in bypasses would limit the BC productivity. Meanwhile, the nucleic acids, proteins, ATP, NADPH, and other molecules weregenerated as well, which could be used as precursors for cell growth and BC production [10]. In our previous study [15], metabolic flux analysis for the central carbon metabolism revealed that about 47.96% of glycerol was transformed into BC, while only 19.05% of glucose and 24.78% of fructose were transformed into BC. Instead, when glucose was used as the sole carbon source, 40.03% of the carbon source was turned into the by-product gluconic acid.

In this study, a mutant strain was obtained by combined chemical mutation, with a high productivity of BC. Using this metabolic network and knowledge of the precursor molecules, metabolic flux distributions were calculated and compared between the BC high-yield mutant and the parent strain *G. xylinus* CGMCC 2955. This information provides a theoretical foundation of metabolism in *G. xylinus* CGMCC 2955 that could inform future genetic manipulations.

## Materials and Methods

### Microorganism

*G. xylinus* CGMCC2955 was screened by the Key Laboratory of Industrial Microbiology, Ministry of Education, Tianjin University of Science and Technology, stored in China General Microbiological Culture Collection Center with the registered number, NO. 2955 [5], [10].

### Culture Medium and Growth Conditions

Pre-cultures were grown in 500 mL baffled shake flasks with 100 mL medium containing 25 g/L glucose, 7.5 g/L yeast extract, 10 g/L peptone, and 10 g/L Na_2_HPO_4_·2H_2_O. The medium was acidified to pH 5.5–6.0 by hydrochloric acid and sterilized at 121°C for 20 min. After culture at 30°C for 24 h, cell suspension was inoculated into other 500 mL baffled shake flasks with chemically defined medium at a ratio of 8% (v/v), static culture at 30°C for 8 days. Chemically defined medium contained glucose (25 g/L), Na_2_HPO_4_ (3 g/L), KH_2_PO_4_ (1 g/L), (NH_4_)_2_SO_4_ (5 g/L), MgCl_2_ (0.02 g/L), CaCl_2_ (0.02 g/L) and para-aminobenzoic acid (PABA) (0.0015 g/L), was used for metabolic flux analysis [10]. The glucose was separately sterilized at 115°C for 15 min.

### Mutagenesis Procedure

Combined chemical mutation was used to mutate the parent strain by DES (diethyl sulfate) with LiCl, intending to obtain a mutant strain that generates a lower quantity of byproducts, specifically gluconic acid and acetic acid. Biomass suspensions that had been treated by DES for different intervals were spread on a plate containing 0.3% (w/v) LiCl. The dose-dependent mutation lethality curve was illustrated by counting of the colonies on the above plate. The lethal dose and screening reagent were 30 min and bromophenol blue (0.4 g/L), respectively. When a colony grows in the petridish after being cultured for 4–5 d, the pH of the medium around the colony will decrease, which would lead to the color change of bromophenol blue (from yellow to blue), the ones that get lager colony and smaller color ring, are supposed to be high yield mutant.

### Analytical Techniques

Acetic acid and gluconic acid were analyzed by a Waters high-pressure liquid chromatography (HPLC) at 230 nm with UV detector, equipped with a Shodex NH2P-50 column (150 mm * 4.6 mm, Showa Denko K.K., Japan). The mobile phase consisted of 0.05 M KH_2_PO_4_ at a flow rate of 1 mL/min.

To separate the cells from the cellulose, 2% (v/v) cellulase (5000 U/mL) was added into medium at the end of the culture, processed 2 h to degrade cellulose and release cells. Cell dry weight was determined using 10 mL cell suspensions in five replicates. Samples were centrifuged at 6,000 rpm, washed twice with 30 mL distilled water, and then dried at 80°C for 10 h to a constant weight. Cellulose production was measured based on at least five replicates, which was collected by soaking and washing the cellulose pellicle with NaOH (0.1 M) to lyse cells, washed by water until pH-neutral and dried at 80°C for 10 h to a constant weight.

### Assay of Enzymatic Activities

The enzymatic activity of phosphoenolpyruvate carboxylase (PEPC) in mutant and parent strains was assayed spectrophotometrically by monitoring the disappearance of NADH at 340 nm (PEPC kit, Nanjing Jiancheng Institute of Biotechnology, Nanjing, China) [18]. The activity of succinate dehydrogenase (SDH) was detected using succinate/DCPIP oxidation-reduction assay kit (Nanjing Jiancheng Institute of Biotechnology, Nanjing, China) [19]. PEPC and SDH activities were both represented as units/OD_600_:
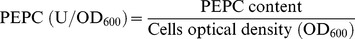


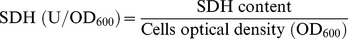



The ATPase activity in parent and mutant strain was determined by measuring the formation of phosphoric acid from ATP [20], [21]. The measurement procedure was referred to the reagent kit instruction (Nanjing Jiancheng Institute of Biological Engineering, Nanjing, China) as follows:

ATPase activity intracellular = (*A_1_*−*A_3_*) ÷ *A_2_* × *C* × *D* ÷ *T* µmol/OD_600_, where *A_1_*, *A_2_* and *A_3_* are the absorbance of sample, standard and control, respectively. *C* is the phosphoric content of standard. *D* is the dilute times of sample and *T* is the optical density of cells.

Cells concentration was measured each day as optical density (OD_600_). Samples were prepared as follows: cultured cells were harvested by centrifugation at 5000 rpm for 5 min at 4°C, 400 µL of extraction solution was added into 2,000,000 cells. Suspension was exposed to ultrasonic cell disruption system (power 20%, ultrasonic 3 seconds, interval 10 seconds, repeated for 30 times). The obtained supernatant was centrifuged at 8000 × g for 10 min at 4°C, and then put in the ice bath before measurement.

### Flux Balance Model

A stoichiometric model combined with extracellular metabolite measurement was applied to the estimation of intracellular fluxes [22], [23], [24]. A bioreaction network with branch point-associated metabolites was assembled to calculate intracellular fluxes. Network reactions in the central metabolism were determined, and the biosynthetic pathway to BC was constructed as described by Ross *et al.* [25] and Tonouchi *et al*. [26]. The reaction network consisted of 23 metabolites and 21 reactions with some unknown fluxes. A (pseudo)-steady-state approximation, in which the sum of the fluxes to and from any particular intermediary metabolite equals to zero, was used to generate the following linear mass balance equation for intracellular metabolite pools, and then calculate the flux distributions:
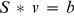
(1)


In this equation, *S* is the matrix of the stoichiometric bioreactions (23*21) based on the metabolic map, *v* is the vector of 21 unknown metabolic fluxes to be determined, and *b* is the vector of 23 known fluxes from measurable products and substrates for each of the 23 metabolite balances. The solution to equation (1) was determined by a constrained least-squares approach with the objective of minimizing the sum of the squares of residuals from the metabolite mass balances. The only constraint in the least-squares problem was that the fluxes be non-negative for irreversible reactions. Here we used LINGO [27], [28] to solve the equation and obtain the values of metabolic fluxes.

## Results

### Obtaining Mutant Strain with High BC Productivity

To reduce the amount of byproducts and improve BC yield, different groups have carried out various methods that are summarized as follows. A mutant strain with high BC yield was obtained by MNNG (*N*-methyl-*N'*-nitro-*N*-nitrosoguanidine) or EMS (ethyl methane sulfonate) mutation [29]. Following this, a glucose dehydrogenase (GDH)-deficient mutant of strain BPR2001, GD-I, was generated via gene disruption using the cloned gene fragment. Strain GD-I did not produce any gluconic acid, but produced 4.1 g/L of BC aerobically using glucose as the carbon source [30]. In another study, a mutant lacking the genes for acetan production was found to have no improvement in cellulose synthesis [31].

**Figure 1:**
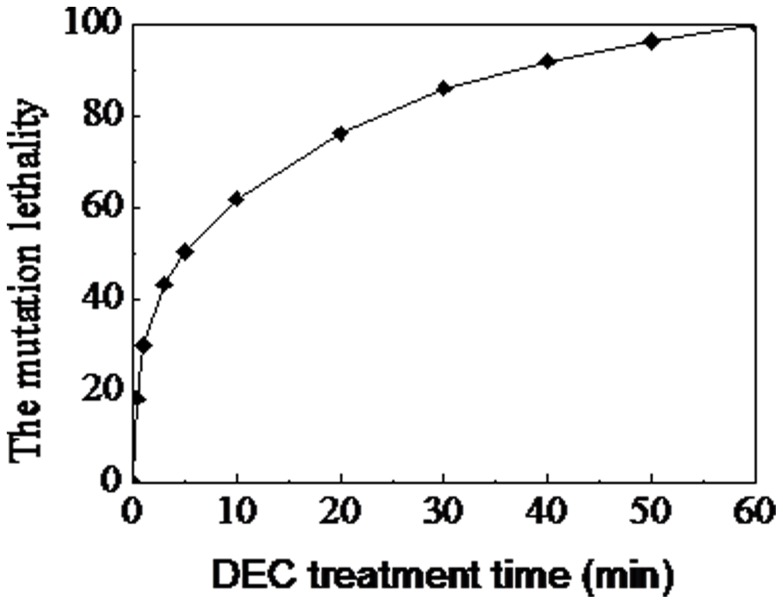
Dose-dependent mutation lethality curve of *G. xylinus* (CGMCC no.2955) for DES-LiCl compound mutation.

In this study, chemical-compound mutation was used to mutate the parent strain by DES (diethyl sulfate) with LiCl. The mutation lethality increased rapidly from 0 to 30 min, but after 30 min the increase of lethality leveled off. Thus, the DES treatment time was set at 30 minutes for which the lethality was 85.9% (Fig. 1).

After 2 rounds of compound mutation, a mutant strain with a high BC yield and relatively high final pH (indicating low production of acid byproducts) was obtained from the parent strain *G. xylinus* CGMCC 2955. The BC production reached 11.75 g/L in the mutant strain (named *G. xylinus* AX2-16); final pH value in culture broth was 4.86, which was higher than that of the parent strain; and gluconic acid concentration was 5.71 g/L, which was about 56% lower than that of parent strain.

**Figure 2:**
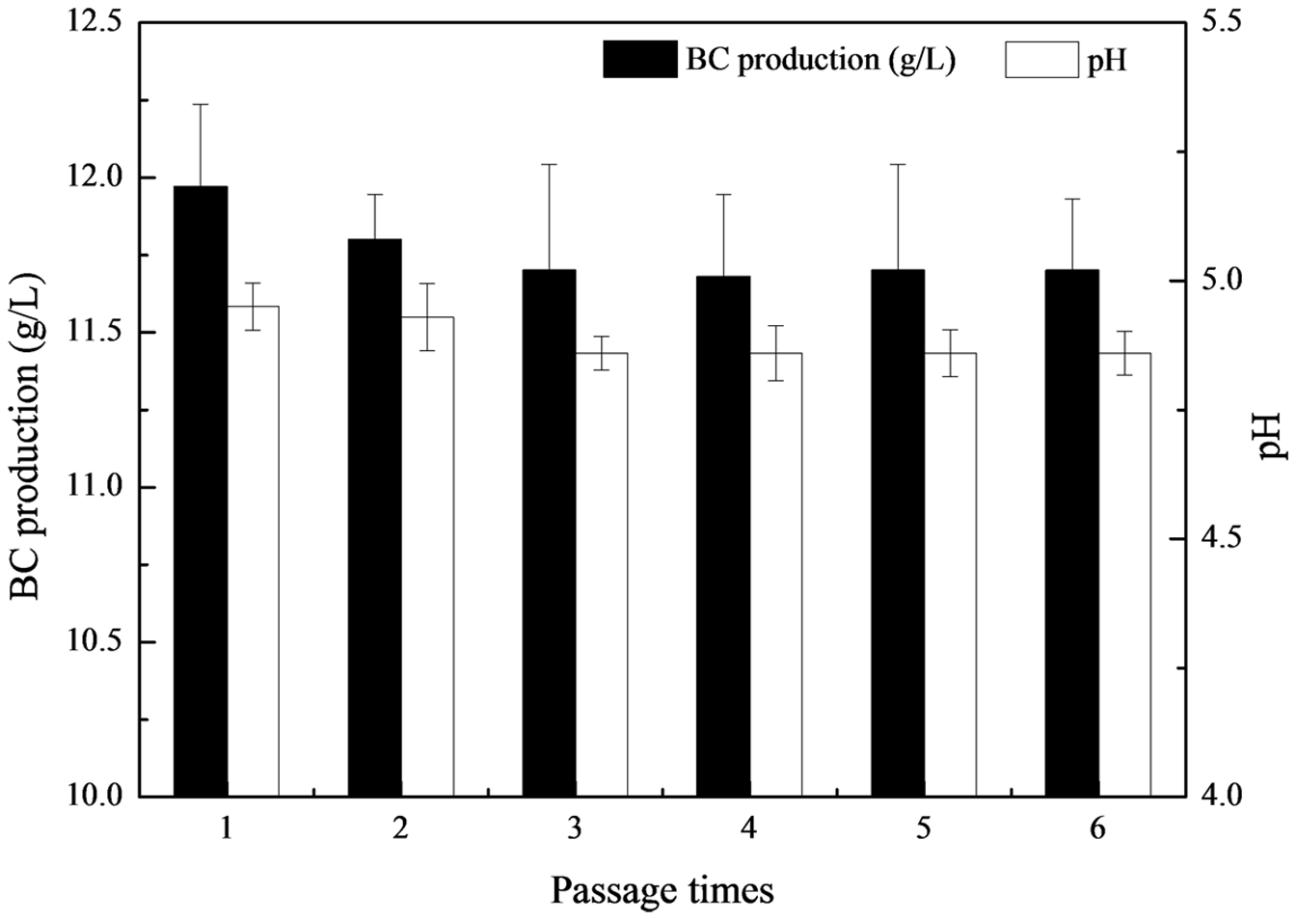
Transgenerational variation in the production of bacterial cellulose and the pH value for mutant strain AX2-16.

In order to determine the genetic stability of the mutant strain, *G. xylinus* AX2-16, the strain was transferred to Erlenmeyer flasks for observation of BC and byproduct generation (estimated by pH) during serial passages (Fig. 2). From passage 1 to 3, the BC production and pH value decreased to 11.75 g/L and 4.86, however the two parameters were stable through all passages following passage 3.

### Metabolic Network Construction of *G. xylinus* (CGMCC No. 2955)

**Figure 3:**
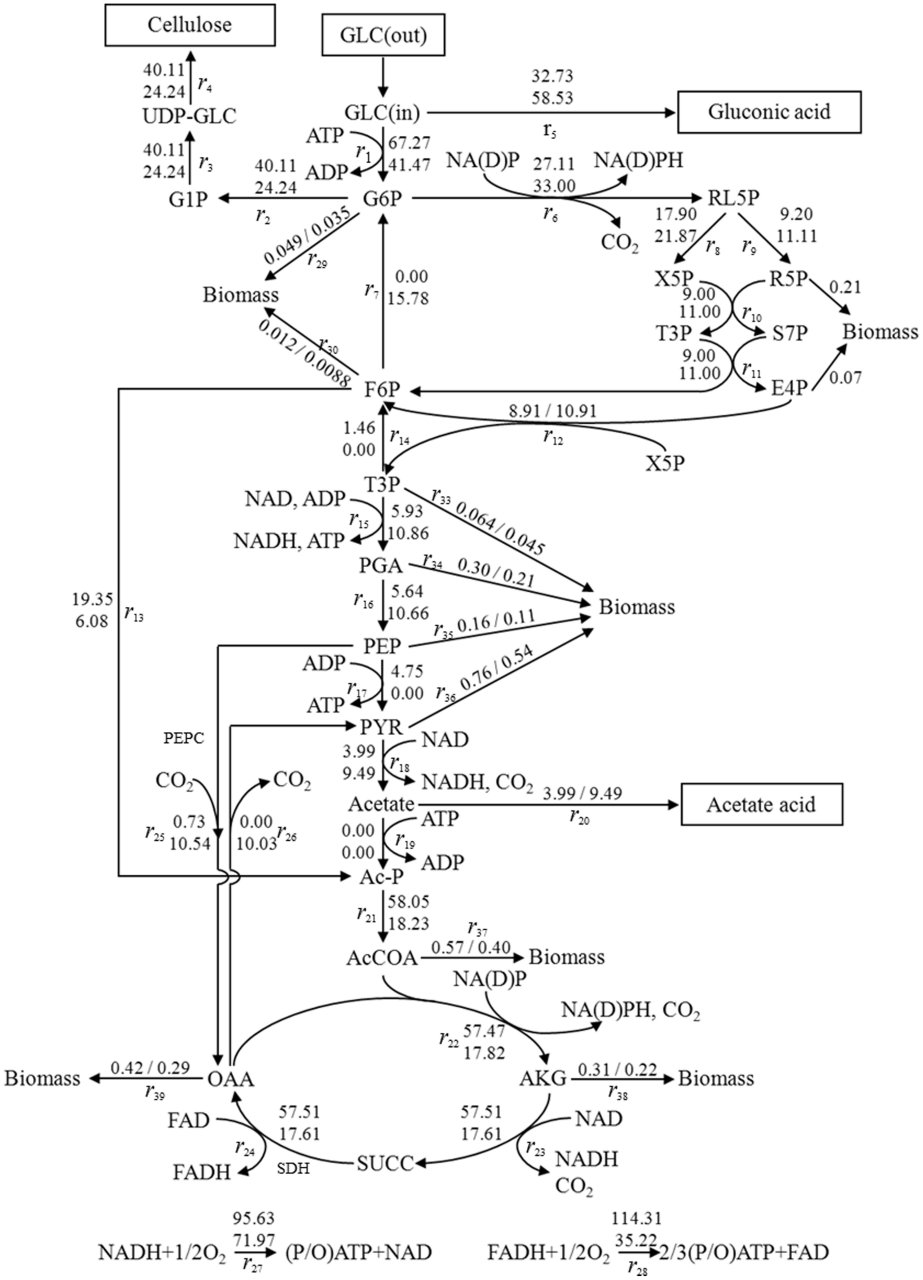
Metabolic network of mutant and parent strains (Upper data- *G. xylinus* AX2-16, lower data- *G. xylinus* CGMCC 2955). Glucose was the sole carbon source (GLC: glucose; G6P: glucose-6-phosphate; G1P: glucose-1-phosphate; UDP-GLC: Uridine diphophoglucose; RL5P: Ribulose-5-phosphate; X5P: Xylulose-5-phosphate; R5P: Ribose-5-phosphate; T3P: Glyceraldehyde-3-phosphate; S7P: Sedoheptulose-7-phosphate; E4P: Erythrose-4-phosphate; F6P: Fructose-6-phosphate; PGA: 3-Phosphoglycerate; PEP: Phosphoenol pyruvate; PYR: Pyruvate; Ac-p: Acety phosphate; AcCoA: Acetyl-coenzyme-A; AKG: α-keto-gluterate; SUCC: Succinate; OAA: Oxaloacetate; PEPC: Phosphoenolpyruvate carboxylase; SDH: Succinate dehydrogenase).

In our recent work [15], we have constructed the reaction network of central carbon metabolism in *G. xylinus* (CGMCC No.2955). It has to be stated that the inability to metabolize glucose under anaerobic condition in *G. xylinus* results from the lack of phosphofructokinase that is required for glycolysis [14], [32]. Hence, flux from F6P to T3P does not exist. Gluconeogenesis occurs in *G. xylinus* CGMCC 2955 from oxalacetate via pyruvate (*r*_18_) due to the unusual regulation of the oxaloacetate decarboxylase and pyruvate phosphate dikinase [33], [34]. As shown in Fig. 3, there were fluxes from phosphoenolpyruvate to oxaloacetate (*r*_25_) and oxaloacetate to pyruvate (*r*_26_) for glycolysis and gluconeogenesis, respectively. Thus, cellulose is produced by *G. xylinus* CGMCC 2955 from a metabolic pool of hexose phosphate that is converted directly by the phosphorylation from exogenous hexoses, as well as indirectly via the pentose cycle and the gluconeogenic pathway. The determined fluxes (*r*_1_, *r*_2_, *r*_3_, *r*_4_) from glucose to cellulose are glucose → glucose 6-phosphate → glucose 1-phosphate → UDP-glucose → cellulose. Other studies have shown that lipid- and protein-linked cellodextrins may function as intermediates between UDP-glucose and cellulose in *G. xylinus* [35].

It was assumed that NADPH was produced only to fulfill biosynthetic requirements, and the NADH flux was assumed to be proportional to the oxygen uptake. The biosynthetic requirements for ribose-5-phosphate, 3-phosphoglycerate, and NAD(P)H were specified as follows. Although the NAD- and NADP-linked dehydrogenases are known to catalyze the same reaction in *G. xylinus*, the NAD-related glucose 6-phosphate dehydrogenase of *G. xylinus* CGMCC 2955 is involved mainly when the pentose-phosphate pathway is directed toward oxidation and energy generation, while the NADP-related enzyme functions in an anabolic capacity. Furthermore, the NAD-specific enzyme rather than NADP-dependent dehydrogenase is sensitive to inhibition by ATP. Therefore, the reactions from glucose 6-phosphate dehydrogenase to ribulose 5-phosphate (*r*_6_) involving NAD or NADP, depend on the different growth conditions. It has also been reported that the malate dehydrogenase of *G. xylinus* is an FAD-enzyme containing an iron-binding site that is essential for its activity [36], hence we specify that this flux (*r*_24_) in our strain is involving FAD.

In *G. xylinus*, since phosphofructokinase, an essential enzyme in the glycolytic pathway, could not be synthesized, the fructose-6-P phosphoketolase pathway may represent a very desirable metabolic feature. Meanwhile, conversion from fructose-6-P to acetate (*r*_13_) produces 3 moles of ATP per mole of fructose-6-P, or 2 moles of ATP per mole of glucose, the yields of which are identical with that of glycolysis. Therefore, a unique pathway (*r*_13_) exists from fructose-6-P to acetate in *G. xylinus* CGMCC 2955.

### Enzymatic Activities

**Figure 4:**
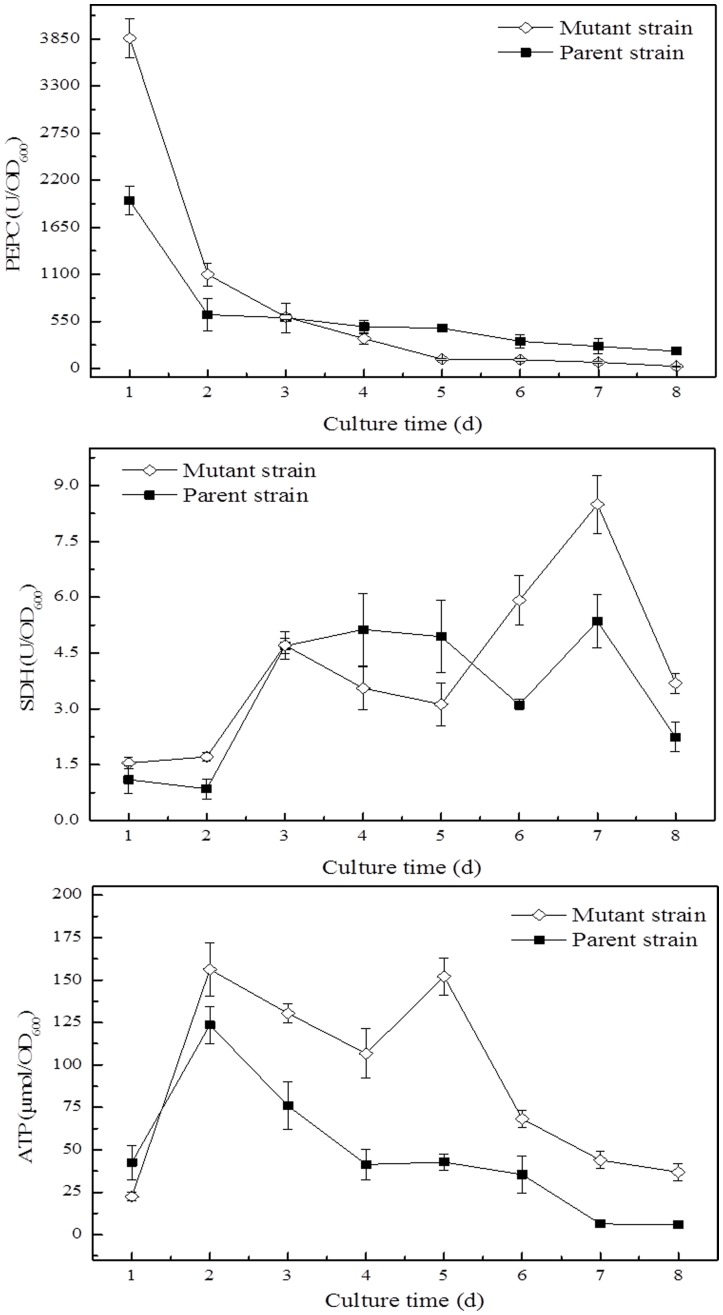
The changes of enzymatic activities of PEPC, SDH and ATPase in mutant and parent strains. The activities of PEPC, SDH and ATPase in mutant and parent strains were detected as described in Materials and methods. Cells were cultured statically at 30°C for 8 days.

In *G. xylinus,* PEPC catalyzes the addition of bicarbonate to phosphoenolpyruvate (PEP) to form the four-carbon compound oxaloacetate (OAA) and inorganic phosphate, corresponding to the bioreaction of *r_25_* in Fig. 3 [37]. As shown in Figure 4, the enzymatic activity of PEPC in mutant strain AX2-16 was 1.97-fold of that in parent strain on the 1^st^ day. As culture time extended, PEPC in *G. xylinus* AX2-16 showed lower activity than that in parent strain. For example, on the 4^th^–8^th^ day, its activity in mutant strain was only 71.60%, 22.91%, 32.20%, 27.65% and 12.19% of that in parent strain, respectively. This result was consistent with the value of *r_25_* from flux analysis. As shown in Figure 3, for *r_25_*, the flux in *G. xylinus* AX2-16 was 0.73, while it was 10.54 in the parent strain *G. xylinus* CGMCC 2955.

SDH is one of the key enzymes in TCA cycle, with its activity tightly correlated with the cellular energy metabolism. It catalyzes the oxidation of succinate to fumarate with the reduction of ubiquinone to ubiquinol [38]. The changes of SDH activity in both strains showed similar trends as a function of culture time. The activity of SDH in *G. xylinus* AX2-16 was shown at high level since the 6^th^ day. It has to be noted that the SDH activity in *G. xylinus* AX2-16 was obtained at 8.50 U/OD_600_ on the 7^th^ day, compared with 5.35 U/OD_600_ in *G. xylinus* CGMCC 2955 at the same time. At the end of culture (8 d), the SDH activity decreased in both the two strains, but the SDH activity in mutant strain still showed 1.65-fold of that in parent strain.

The ATPase activity in *G. xylinus* AX2-16 exhibited a much higher value than that in *G. xylinus* CGMCC 2955 since the 2^nd^ day. Higher ATPase activity in *G. xylinus* AX2-16 was obtained at 152.08 µmol/OD_600_ on the 5^th^ day, which was almost 3.56-fold of that in parent strain. As culture proceeded, the ATPase activities in both strains decreased. However, at the end of culture, the enzymatic activity of ATPase in *G. xylinus* AX2-16 was still 6.41-fold of that in *G. xylinus* CGMCC 2955.

## Discussion

### Comparison of Cultivation Process between *G. xylinus* (CGMCC No. 2955) and AX2-16 in Batch Culture

**Figure 5:**
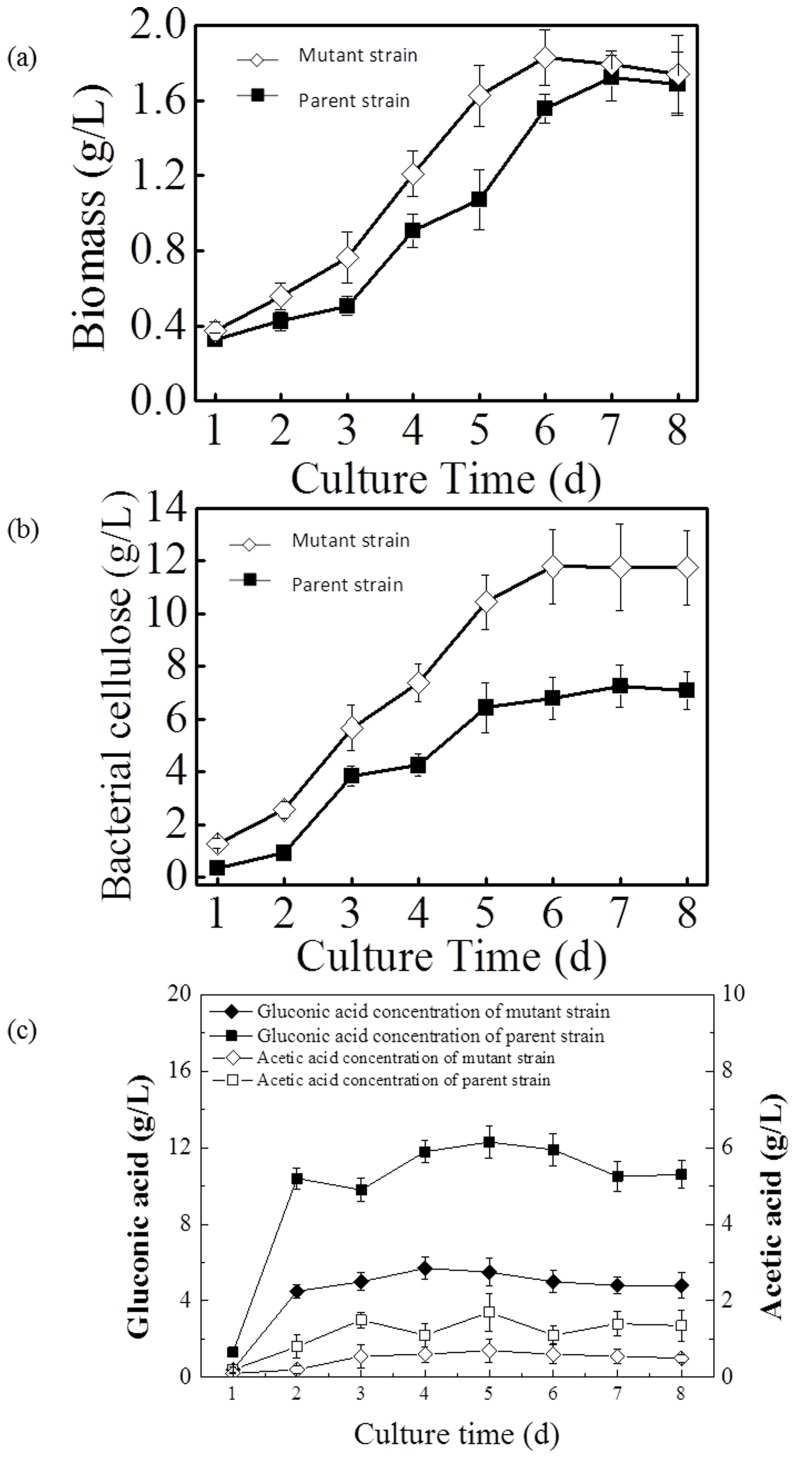
Changes in biomass, gluconic acid, and bacterial cellulose production during batch culture using glucose as the sole carbon source. (a) biomass, (b) bacterial cellulose, (c) gluconic acid.

Batch cultures of the parent strain *G. xylinus* CGMCC 2955 were conducted to determine the growth parameters using glucose as the sole carbon source. BC, gluconic acid, and biomass production in *G. xylinus* CGMCC 2955 were measured. Fig. 5 shows that for the parent strain, the maximum production of BC (7.26 g/L) and dry biomass accumulation (1.72 g/L) were observed on the 7^th^ day. As shown in Fig. 3, the biomass concentration of *G. xylinus* AX2-16 was always higher than that of *G. xylinus* CGMCC 2955 during the cultivation process. Particularly, at the exponential phase from 3^rd^ to 5^th^ day, the biomass accumulation of *G. xylinus* AX2-16 was about 1.5 times higher than that of *G. xylinus* CGMCC 2955 (Fig. 5a). It was interesting to find that the gluconic acid concentration of AX2-16 was only half of the parent strain’s (Fig. 5c). Final BC production from *G. xylinus* AX2-16 was also 62% greater than *G. xylinus* CGMCC 2955 (Fig. 5b). Correspondingly, the BC productivity by *G. xylinus* AX2-16 was obtained at 2.75 mmol/g·h, which was 1.34 fold of that in *G. xylinus* CGMCC 2955 (Table 1).

**Table 1:** Summary of Fermentation Parameters in Batch Culture by Parent strain and Mutant Strain.

Strain	Glucosemmol/g.h	BC productivitymmol/g.h	gluconic acidmmol/g.h	acetic acidmmol/g.h	Dry biomassconcentrationg/L	µ(Specific growth rate)h^−1^
*G. xylinus*(CGMCC no 2955)	8.45±0.56	2.05±0.11	4.94±0.34	0.80±0.04	0.91±0.06	0.014±0.002
*G. xylinus AX 2-16*	6.85±0.41	2.75±0.18	2.24±0.15	0.27±0.02	1.21±0.07	0.016±0.001

### Comparison of Metabolic Flux Distributions in the Mutant and Parent Strain

Chemical mutagenesis is a random method to alter microorganisms. In other words, genetic or metabolic changes of microorganisms in this process were unpredictable. However, by estimating metabolic fluxes based on stoichiometric models, it provides a valuable and convenient tool to reveal the metabolic distributions in the parent and mutant strains. A stoichiometric mass balance analysis was used to give a quantitative description of the flux distribution within the defined bioreaction network, as shown in Fig. 3. In this network, the quantity of glucose that entered the cell was normalized to 100. For the parent strain, almost 59% and 9.5% of carbon was directed to gluconic acid and acetic acid, respectively, which were regarded as byproducts in the generation of BC, and a waste of the carbon source. Only 24% of the carbon source converted to the final product, BC. Therefore, the key objective of the induced mutation was to increase the efficiency of converting glucose to BC.

It was shown that 1.7% and 2.4% of the source carbon (derived from glucose) was used to generate cell biomass for *G. xylinus* CGMCC 2955 and *G. xylinus* AX2-16, respectively. The flux of carbon source to the desired end-product, BC in parent strain increased to 40%, compared to 24.2% of the parent strain. In addition, the carbon flux to the byproduct, gluconic acid, decreased for *G. xylinus* AX2-16 at only 32.7%, compared with 58.5% of that in the parent strain *G. xylinus* CGMCC 2955. The fraction of source carbon that ended in the secondary byproduct, acetic acid, was fairly similar for parent and mutant, with 9.5% and 4.0%, respectively. Within the metabolic pathway of *G. xylinus* CGMCC 2955, 33.0% of the carbon source (glucose) entered into the pentose phosphate pathway (PPP) (*r*_6_) and approximately 17% entered into TCA cycle (*r*_22_, *r*_23_ and *r*_24_) (Fig. 3). However, for strain *G. xylinus* AX2-16, a dramatically higher TCA cycle flux (57.0%) was obtained. Bass *et al.* reported that the enzyme activities within each metabolic pathway are closely related [39]. As shown in Figure 4, the enzymatic activity of SDH from *G. xylinus* AX2-16 was 1.90-fold, 1.59-fold and 1.65-fold higher than that from parent strain on the 6^th^, 7^th^ and 8^th^ day, respectively. This confirmed the flux analysis result that higher TCA cycle activity was obtained in mutant strain than that in parent strain. Furthermore, the PPP was the primary metabolic pathway through which glucose was able to enter TCA cycle, which also requires additional ATP. TCA cycle is a series of chemical reactions to generate energy (in the form of ATP) through the oxidation of acetate. The relatively high level of TCA cycle flux may also be explained by higher ATP requirements for biosynthesis of cellulose. As illustrated by Fig 4, the ATPase activity in *G. xylinus* AX2-16 was much higher than that in the parent strain after the 2^nd^ day. It has to be noted that the enzymatic activity of ATPase in *G. xylinus* AX2-16 was still 6.41-fold of that in *G. xylinus* CGMCC 2955 at the end of culture, indicating that more ATP was demanded in mutant strain AX2-16 compared with parent strain. For strain *G. xylinus* CGMCC 2955, 15.8% glucose returned to glucose-6P, while the reversible reactions between glucose-6P and fructose-6P remained balanced in the *G. xylinus* AX2-16, thereby, reducing the generation of gluconic acid. Moreover, for strain *G. xylinus* AX2-16, the majority of the flux entered into F6P and then into Ac-P and TCA cycle in order to produce energy. A number of fluxes for *G. xylinus* AX2-16 were equal to zero (*r*_7_, *r*_17_ and *r*_19_). For *r*_7_, this flux may be zero if strain AX2-16 does not have the G6P recycle to produce F6P. For *r*_17_, the parent strain probably requires more carbon source for gluconeogenesis (PYR to PEP, the major bioreaction of gluconeogenesis) and from there to gluconic acid and BC. For *r*_19_, some Ac-P was spontaneously hydrolyzed to phosphorus and acetate. Finally, for *r*_13_, the flux in strain AX2-16 was about 3.5 times of that in *G. xylinus* CGMCC 2955, indicating that there was more flux into the Ac-P, and then into the TCA cycle to produce more ATP for biosynthesis in the mutant strain.

Generally, NADPH formation is strictly coupled to the biosynthetic NADPH requirements, which causes the PPP to operate only to satisfy the cellular requirements for NADPH and pentose precursors. However, in *G. xylinus*, the operation of the pentose phosphate pathway is not exclusively governed by the demand for NADPH and pentose precursors. In phosphoglucoisomerase-negative mutants of *E. coli*, glucose cannot be metabolized via glycolysis, but only through the PPP [40]. Instead, for these mutants, G6P is metabolized by pentose-phosphate (PP) and phospho-ketolase (PK) pathways. These pathways are linked to the intracellular acyl-phosphate pool [41]. In addition to anabolic utilization of NADPH, *G. xylinus* could also reoxidize to NADH. PPP flux is regarded to be in the range of 20% to 30% of the total glucose uptake in bacteria, which exceeds the requirements for NADPH and pentose formation under most conditions [42]. These bacteria also could use NADH produced in the TCA cycle via G6P dehydrogenation, and NADPH produced in the PP pathway [43]. Other bacteria also have been shown to produce NADPH in excess of their biosynthetic requirements for NADPH. Using labeling studies combined with metabolite balancing, the actual NADPH formation in lysine-producing *Corynebacterium glutamicum* was found to exceed the biosynthetic requirements by 21.1% [44]. Similar to the mutant *E. coli* above, *G. xylinus* CGMCC 2955 lacked phosphofructose kinase required for glycolysis, and the glucose was only metabolized by PPP flux by 27-33% of the glucose. Hence, as the pentose phosphate pathway is necessary for conversion of G6P to F6P in *G. xylinus* CGMCC 2955, constraining the flux through this pathway to the amount required solely for NADPH requirements, may lead to a biased solution, in which all of the substrate carbon not in biomass and products will be converted to CO_2_ in the TCA cycle. In order to obtain an unbiased estimation of the flux pattern, it was assumed that interchangeability of reducing equivalents, NADH and NADPH, could be explained biologically by the presence of a transhydrogenase [42]. Based on this assumption, the estimated oxidative PPP flux was adjusted and reached relative fluxes higher than unity at the expense of the TCA cycle. If no further assumptions regarding the biological function of the PPP are made under these conditions, the estimated PPP flux is very sensitive to the respiratory quotient. The best estimate was obtained by allowing a realistic 20% exchange of reducing equivalents from NADPH to NADH in the optimized calculations, based on the experimental results of Marx *et al.* [44]. The flux estimates obtained with this assumption for the PPP and TCA cycles indicated the robustness of the solution for a transfer of reducing equivalents from NADPH to NADH in the physiological range. Therefore, the NADPH and NADH are considered as equivalents. In another words, all of the NADPH overproduced by the PPP will be converted into NADH and completely oxidized in the TCA cycle.

Using chemical mutation by DES and LiCl, a mutant strain, *G. xylinus* AX2-16, was obtained with highest BC productivity of 11.75 g/L. However, gluconic acid, the main byproduct, was only produced at 5.71 g/L by mutant strain, which was 55.7% lower than that of parent strain. Metabolic flux analysis demonstrated that 40.1% of the source carbon was diverted into the desired product BC in mutant strain, compared with 24.2% for parent strain. Additionally, only 32.7% of carbon source was fluxed into gluconic acid in mutant strain, compared with 58.5% of that for parent strain. A higher flux of TCA cycle was obtained in mutant strain (57.0%) compared with parent strain (17.0%), which matched well with the results from enzymatic analysis. It indicated that the increased TCA cycle flux and ATP content in mutant strain would be attributed to the acceleration of BC biosynthesis.
